# Molecular Analysis of a Genetic Variants Panel Related to Nutrients and Metabolism: Association with Susceptibility to Gestational Diabetes and Cardiometabolic Risk in Affected Women

**DOI:** 10.1155/2017/4612623

**Published:** 2017-01-04

**Authors:** Marica Franzago, Federica Fraticelli, Antonio Nicolucci, Claudio Celentano, Marco Liberati, Liborio Stuppia, Ester Vitacolonna

**Affiliations:** ^1^Laboratory of Molecular Genetics, Department of Psychological, Health and Territorial Sciences, School of Medicine and Health Sciences, “G. d'Annunzio” University, Chieti-Pescara, Via dei Vestini 31, 66013 Chieti, Italy; ^2^Ce.S.I-Met, “G. d'Annunzio” University, Chieti-Pescara, Via Colle dell'Ara No. 1, 66100 Chieti, Italy; ^3^Department of Medicine and Aging, School of Medicine and Health Sciences, “G. d'Annunzio” University, Chieti-Pescara, Chieti, Italy; ^4^Center for Outcomes Research and Clinical Epidemiology (CORE), Pescara, Italy

## Abstract

Gestational diabetes mellitus (GDM) is the most frequent metabolic disorder in pregnancy. Women with a GDM history are at increased risk of developing diabetes and cardiovascular diseases. Studies have demonstrated a significant correlation between several genes involved in the metabolic pathway of insulin and environmental factors. The aim of this study was to investigate the relationship between clinical parameters in GDM and variants in genes involved with nutrients and metabolism. Several variants* PPARG2* rs1801282 (*C>G*);* PPARGC1A* rs8192678 (*C>T*);* TCF7L2* rs7903146 (*C>T*);* LDLR* rs2228671 (*C>T*);* MTHFR* rs1801133 (*C>T*);* APOA5* rs662799 (*T>C*);* GCKR* rs1260326 (*C>T*);* FTO* rs9939609 (*T>A*);* MC4R* rs17782313 (*T>C*) were genotyped in 168 pregnant Caucasian women with or without GDM by High Resolution Melting (HRM) analysis. A significant correlation was observed between TT genotype of* TCF7L2* gene and increased risk of GDM (OR 5.4 [95% CI 1.5–19.3]). Moreover, a significant correlation was observed between lipid parameters and genetic variations in additional genes, namely,* PPARG2* [*p* = 0,02],* APOA5* [*p* = 0,02],* MC4R* [*p* = 0,03],* LDLR* [*p* = 0,01], and* FTO* [*p* = 0,02]. Our findings support the association between* TCF7L2* rs7903146 variant and an increased GDM risk. Results about the investigated genetic variants provide important information about cardiometabolic risk in GDM and help to plan future prevention studies.

## 1. Introduction

Gestational diabetes mellitus (GDM) is defined as “diabetes diagnosed in the second or third trimester of pregnancy that is not clearly overt diabetes” [1].

GDM approximately affects 7% (range 2–18%) of all pregnancies [2] and may result in short- and long-term complications for both mother and foetus/infant [3], and GDM can be considered an unmasking of underlying and silent risk of diabetes and cardiovascular disease. Although the etiology of this disease has not been fully elucidated, overweight status before pregnancy, weight gain during pregnancy, ethnicity, and family history of diabetes play important roles in the development and progression of GDM [4]. Moreover, previous studies have demonstrated a significant correlation between polymorphisms of several genes involved in the metabolic pathway of insulin and increased risk for GDM [5, 6].

The recent advances in molecular technology have underscored the significant role of genetic factors in the development, treatment, and complications of diabetic pregnancy [7]. However, so far the only example of genetic predisposition to GDM is represented by maturity onset diabetes of the young (MODY), a clinically heterogeneous autosomal dominant monogenic disease, accounting for up to 5% of diabetes and due to mutations in different genes such as* HNF4A*,* GCK*,* HNF1A*,* IPF1*,* HNF1B*, and* NEUROD1*. Common variants in MODY genes, especially in* HNF1A *and* GCK,* have been correlated with GDM and related traits [8–10].

The identification of additional genetic markers which could explain differences in susceptibility to GDM would represent a crucial point in order to set up a strategy for the prevention, early diagnosis, and treatment of this condition. In this regard, the aim of this study was to investigate the relationship between clinical parameters in GDM and variants in genes involved with nutrients and metabolism (namely,* TCF7L2*,* PPARG2*,* PPARGC1A*, carbohydrate metabolism;* FTO*,* MC4R*,* APOA5*,* LDLR*,* GCKR*, fat metabolism;* MTHFR*, folate metabolism). These variants were selected due to their correlation with lipid metabolism, glucose sensing, beta-cell function, and appetite control and are all adequately replicated by the literature. An additional aim was to identify an innovative tool for early prevention of cardiometabolic diseases after GDM. To the best of our knowledge, this is the first association study in GDM based on the use of a genetic panel able to identify variants related to the metabolism of both carbohydrates and lipids, in order to evaluate not only the susceptibility to GDM, but also the increase in cardiometabolic risk.

## 2. Materials and Methods

### 2.1. Study Design and Participants

From February 2015 to November 2015, one hundred and sixty-eight Caucasian women attending the Diabetes and Metabolism Unit or the Obstetrics and Gynaecology Clinic at the Hospital of University “Gabriele d'Annunzio” in Chieti were recruited. Specifically, 102 consecutive women with GDM (mean age ± SD = 34.6 ± 5.4) and 66 nondiabetic pregnant consecutive control subjects (mean age ± SD = 31.9 ± 5.1) were assessed. The diagnosis of GDM was based on an abnormal 75 g oral glucose tolerance test (OGTT) where either one or more blood glucose values were above the values of 5.1 mmol/L (92 mg/dL) at fasting, 10.0 mmol/L (180 mg/dL) at 1 h, and 8.5 mmol/L (153 mg/dL) at 2 h. [11]. The genetic analysis was conducted at the Laboratory* of Molecular Genetics, School of Medicine and Health Sciences, *“G. d'Annunzio” University of Chieti.

### 2.2. Methods

During the first visit (conducted at the Diabetes and Metabolism Unit or at the Obstetrics and Gynaecology Clinic) data on sociodemographic characteristics, anthropometric and clinical parameters were collected. In addition, clinical parameters, including prepregnancy body mass index (BMI), gestational weight gain, fasting insulin (FINS), blood glucose, total cholesterol (TC), high-density lipoprotein cholesterol (HDL-C), low-density lipoprotein cholesterol (LDL-C), triglycerides (TG), and blood pressure, were recorded. A blood sample was obtained from each patient and stored at 4°C before DNA extraction.

### 2.3. Inclusion and Exclusion Criteria

The inclusion criteria were pregnant women with ≥18 years of age. The exclusion criteria were women with type 1 or 2 prepregnancy diabetes, overt diabetes, or monogenic diabetes, specifically GCK diabetes, and women with other chronic diseases.

#### 2.3.1. Women with GDM

The diagnostic criteria used for GDM were those proposed by the International Association of Diabetes and Pregnancy Study Groups (IADPSG) [11]. The GDM diagnosis was considered to be proved when established both at the 16–18th and the 24–28th weeks of gestation.

#### 2.3.2. Women without GDM

Women, attending the Diabetes and Metabolism Unit and/or c/o the Department of Obstetrics and Gynaecology, that according to current recommendations [11] had no diagnosis of GDM, were included.

### 2.4. Genotyping

Nine single nucleotide polymorphisms (SNPs) from 9 genes were included in the analysis. Of the 9 genes, three (*TCF7L2*,* PPARG2*, and* PPARGC1A*) were related to carbohydrate metabolism, two (*APOA5*,* GCKR*) to triglycerides metabolism, one (*LDLR*) to cholesterol metabolism, one (*MTHFR*) to folate metabolism, and two (*FTO*,* MC4R*) to energy metabolism.

The SNPs rs1801282 (*C>G*) in* PPARG2*; rs8192678 (*C>T*) in* PPARGC1A*; rs7903146 (*C>T*) in* TCF7L2*; rs2228671 (*C>T*) in* LDLR;* rs1801133 (*C>T*) in* MTHFR* were genotyped. In addition rs662799 (*T>C*) in* APOA5*; rs1260326 (*C>T*) in* GCKR*; rs9939609 (*T>A*) in* FTO*; rs17782313 (*T>C*) near* MC4R* were genotyped. Genomic DNA was automatically isolated from peripheral blood lymphocytes using MagPurix 12s Automated Nucleic Acid Purification System (Zinexts Life Science Corp., Taiwan). SNPs were detected by High Resolution Melting (HRM) technique, according to the manufacturer's instructions. Primers were designed using Primer3 software to amplify a small fragment avoiding the presence of other sequence variations in the primers region. PCR amplification was performed under the same conditions in a 96-well plate in the PikoReal™ Real-Time PCR System (Thermo Scientific). Reaction volume was 10 *μ*L: 1 *μ*L of genomic DNA (10 ng/*μ*L) was added to 9 *μ*L of reaction master mix consisting of Luminaris Color HRM Master Mix (2x) containing chemically modified Hot Start Taq DNA Polymerase, EvaGreen™ fluorescent dye, dNTPs and MgCl_2_ in an optimized PCR buffer with a blue dye, and 0.5 *μ*M of forward and reverse primers. The PCR program started with an initial denaturation of 10 min at 95°C and continued with 40 cycles of 10 s at 95°C, 30 s at 60°C, and 30 s at 72°C: afterward one step for heteroduplex formation by heating to 95°C for 30 s and cooling down to 50°C for 30 s. Data were acquired over the 60–95°C range at the thermal transition rate of 0.2°C/s. Each sample was run in triplicate and each plate contained known controls. Genotypes were assigned by comparing sample curves with control curves. To evaluate the sensitivity of HRM, some of the results were randomly confirmed by direct sequencing.

Moreover, the promoter and the complete coding region for isoform 1 of* GCK* gene was sequenced in 13 pregnant women with suspect MODY2. In detail, fragments containing the promoter and ten exons were amplified by PCR using specific primers designed based on the reference gene sequence. PCR fragments were sequenced using the BigDye Terminator v3.1 and then analyzed on an automatic sequencing analyzer (ABI PRISM 3130XL).

### 2.5. Statistical Analysis

Statistical analysis was performed using the Statistical Package for Social Science (SPSS) program, version 17.0 software for Windows (SPSS, Chicago, IL, USA). *p* values < 0.05 were considered statistically significant. Fisher's exact test or the Chi-square test were used for categorical variables.

The association between gene variants and risk of GDM was assessed through logistic regression analysis with backward variable selection and gene variants: age and BMI tested as covariates. Results are expressed as odds ratios (ORs) with their 95% confidence intervals (95% CI). Comparisons among GDM group to test the effect of carrier/noncarrier and genotypes on TC, HDL-C, and LDL-C levels at 3rd trimester were performed through Mann–Whitney *U* Test and Kruskal-Wallis test, respectively. The association between carrier status and TC, HDL-C, and LDL-C levels at 3rd trimester was also explored through multiple regression adjusted for age and BMI and expressed as *β* parameters. Moreover, comparisons for both GDM and controls to test the effect of carrier/noncarrier and genotypes on BMI were performed through Mann–Whitney *U* Test and Kruskal-Wallis test, respectively. For each investigated locus, Hardy-Weinberg equilibrium was calculated.

## 3. Results

The study of* GCK* gene evidenced the presence in four patients of the variant −30 G>A in the promoter (two in homozygous and two in heterozygous). These cases were excluded from the study.

The characteristics of cases and controls are summarized in Table 1. GDM patients showed higher prepregnancy BMI, age, and lower diastolic blood pressure than control group. This could be because the women with GDM were followed up by the diabetes and nutrition team. All participants underwent OGTT, and mean glucose values according to GDM status are presented in Table 2. The genotype distributions of SNPs in the studies are shown in Supplementary Table  1 (see Supplementary Material available online at https://doi.org/10.1155/2017/4612623.

Only one out of the nine investigated gene variants, namely, rs7903146 in the* TCF7L2* gene, showed a significant difference in the frequency between cases and controls (supplementary Table  1). In fact, the* TT* genotype was significantly more frequent among GDM patients as compared with healthy controls (27,5% versus 10,6%; *p* = 0.024) (supplementary Table  1). At analysis, the* TT* genotype was associated with a more than fivefold increased risk of GDM (OR 5.4 [95% CI 1.5–19.3]). Although the rs7903146 in the* TCF7L2* was the only variant associated with GDM, a significant relation was also found between* APOA5* −1131*T>C *polymorphism, and* MC4R* rs17782313 polymorphism with 3rd trimester HDL-C in women with GDM. Then, a significant correlation was observed between* PPARG2* rs1801282 and* LDLR *rs2228671 with 3rd trimester LDL-cholesterol levels. Finally, a significant correlation was observed between* FTO* rs9939609 and 3rd trimester LDL-cholesterol levels (Supplementary Tables  2-3). Our results show that the associations between SNPs and BMI are not statistically significant (Supplementary Tables  4-5).

After adjusting for age and BMI, the significant association was still present between *C* carrier in* APOA5 *and HDL-C (*β* = −8.6;* p* = 0,01);* T* carrier in* LDLR* and LDL-C (*β* = −43.0;* p* = 0,001);* A* carrier in* FTO* and LDL-C (*β* = 32.4; *p* = 0.017), respectively.

All the investigated genotype frequencies were within the Hardy–Weinberg equilibrium (*χ*^2^ test *p* value > 0.05) both in cases and in controls, except for* TCF7L2 *rs7903146 (*C<T*) and* FTO* rs9939609 (*T>A*) in cases.

## 4. Discussion

In the present work, we evaluated the association between GDM and genetic variants involved with nutrients and metabolism. Results support a significant association between the* TCF7L2* rs7903146 variant and GDM, since the risk was more than fivefold in the* TT* genotype.

The* TCF7L2* gene, located on chromosome 10q25.3, is an important, ubiquitously expressed transcription factor in the Wnt signaling pathway, involved in the proliferation of pancreatic *β*-cells, and controlling the production of incretins such as glucagon intestinal peptide (GIP) and glucagon-like peptide 1 (GLP-1) in intestinal endocrine cells.

The presence of the* TCF7L2 T* allele has been associated with an increased risk of T2DM. In fact, Grant et al. [12] reported that several SNPs within intronic regions of* TCF7L2*, including the rs7903146 (*C>T*), show significant associations with T2DM, and these findings have been replicated by numerous groups, demonstrating the rs7903146 (*C>T*) SNP as one of the most important T2DM susceptibility variants. Dahlgren et al. [13] suggested that the increased risk of T2DM in T allele carriers is associated with dysfunction in the production of insulin within the beta cells and Lyssenko et al. [14] showed that the presence of the *T* allele results in overexpression of* TCF7L2* in the pancreatic *β* cell, with a reduced insulin secretion in turn increasing hepatic glucose production. In addition, several studies previously observed a strong association between this variant and GDM in different ethnic groups [15–22]. Thus, our results confirm the association between rs7903146 (*C>T*) and GDM, previously reported by the literature. In addition, our results demonstrated that this association is not mediated by the presence of variants within other genes involved in food metabolism, among those selected, since no one of the other genes investigated in our study was associated with GDM. Future study will need to investigate the possible role of other genes in this condition.

Nevertheless, our study evidenced also a significant correlation between common genetic variations in several genes (*PPARG2*,* APOA5*,* MC4R*,* LDLR*, and* FTO*) and lipid parameters within the GDM women group.

In this regard, it has been demonstrated that higher level of LDL-C and lower level of HDL-C, as a result of a combination of genetic factors and gene-diet interaction, are directly associated to the risk of CAD or metabolic syndrome. The genetic variations underlying the link between lipids parameters and cardiometabolic traits have been analyzed by various studies but remain unclear.* PPARG2* rs1801282 polymorphism has been associated with impaired insulin sensitivity and was called indeed “insulin resistance locus” [23].

As to the* APOA5*-1131*T>C *variant, interestingly an Italian study based on 1,864 patients <45 years old suggested that this polymorphism may affect the risk of early-onset MI, with an odds ratio of 1.44 (CI: 1.23–1.69) per *C* allele [24]. Gene variants in* MC4R* and* FTO* are associated with severe obesity and metabolic impairment in Caucasians [25]. In the DPS, in men with Impaired Glucose Tolerance the* AA* genotype of rs9939609 in* FTO* was associated with 2.09-fold risk of CVD in men [26].

Regarding our results about the significant correlation observed between* TT *genotype of* LDLR* rs2228671 polymorphismand 3rd trimester LDL-cholesterol levels, it is interesting to note that a recent meta-analysis has established rs2228671 as a protective factor of CHD in Europeans [27].

All these findings are of great relevance and the presence of these gene variants in our panel could increase the use of genetic information in clinical practice for GDM patients: several data support the potential cardiometabolic risk in later life in patients with previous GDM. In fact, Mai et al. [28] demonstrated that GDM increases risk of developing cardiovascular (CV) diseases. Previously, Carr et al. [29] have analysed, in a large population-based retrospective study, the long-term adverse CV outcomes in women with prior GDM. Gestational diabetes is associated with a clustering of cardiovascular risk factors, but the mechanism remains unclear. In this view, the authors have proposed the necessity of new markers to be used to study the effect of GDM on cardiovascular risk during follow-up [29, 30]. Sokup et al. [31] showed that the TG/HDL-C ratio, an early marker of endothelial dysfunction and CV risk, was higher in GDM women one year after the pregnancy, as compared to controls. In this context, of particular relevance is the association found in the present study between, respectively,* APOA5*-1131*T>C* and* MC4R *rs17782313 with 3rd trimester HDL-C in GDM women. This result evokes the study [30] which has shown that an index of early vascular disease (*mean common carotid* IMT-CCIMT) was higher in subjects without diabetes but with previous GDM (pGDM) than in control subjects. In this study when these variables were introduced in a multiple regression analysis, only oxidized LDL (oxLDL) remained significantly associated with CC-IMT. The increased carotid IMT, a marker of preclinical atherosclerosis [32], shows that young women with pGDM have an early sign of vessel involvement, although within the upper limit of normal. These alterations were present in spite of the fact that no woman had diabetes [30].

Furthermore, Corella et al. [33] demonstrated that the* APOA5*-1131*T>C* SNP modulates the effects of macronutrient intake (total fat, carbohydrate, and protein) on BMI and obesity risk in both men and women. In fact, the presence of at least one* C *allele implicated less weight on a high fat diet than the presence of homozygosity for the* T* allele. Future studies are needed to evaluate this aspect and to predict increased cardiovascular events risk and diabetes in the mother and offspring [34].

As is known the diagnosis of GDM identifies a population at high risk for T2DM [35] and metabolic syndrome [30, 36]. The early identification of subjects who could benefit from preventive strategies is a priority for public health. The diagnosis of GDM increasingly represents an exceptional chance to alter the natural course of disease: it is important to have reliable predictive tools. Recently Köhler et al. [37] proposed a novel approach to calculate the diabetes risk in women with prior GDM. In the final predictive risk score were included several variables, as insulin treatment during pregnancy, family history of diabetes, BMI in early pregnancy, lactation, and maternal age at delivery [37]. However, this study did not evaluate metabolic measurements or genetic markers.

GDM represents an important opportunity in the era of “Precision Medicine.” Further studies are required to examine whether incorporation of our panel genes into an algorithm including genetic, clinic, and metabolic variables will help further improving the identification of women with GDM at early risk of diabetes and CHD; this would allow a better allocation of resources and the implementation of effective and sustainable strategies of primary prevention. In the last decades, the nutrition science is taking off in the prevention of noncommunicable chronic diseases that account for more than 60% of global deaths annually [38] and the future goal is the development of personalized genomic medicine. It is necessary understanding the roles of the nutrition and lifestyle matching with genetic and clinic factors in the manifestation of the diseases and its complications. Furthermore, this approach could lead to a reduction in costs of the treatment of nutrition/lifestyle-related chronic diseases [39]. These results are promising, but other functional studies are needed to confirm their biological significance.

The present study has some limitations. First, the pooled sample size for the SNPs was relatively small. Future genome-wide studies with larger sample size are still required to identify genes with smaller allele effects and possibly GDM specific risk variants: this to understand the role of these variants of genes involved with nutrients and metabolism, in the aetiology and progression of GDM. Second, our results show the role of lipids only in GDM because in control group they were unavailable: our study suggests that in the future these parameters should be considered at least during 3rd trimester of pregnancy.

Third, additional analyses, such as epigenetic studies, will also be required to elucidate the role of the molecular mechanisms to the disease susceptibility. In fact, as is known, the development of noncommunicable chronic diseases is determined not only by genetic variants but also by epigenetic changes as DNA methylation and histone modifications, in response to diet and environmental conditions. It has become clear that adverse epigenetic effects might also influence the foetus during pregnancy and postnatal early life increasing the susceptibility to chronic diseases such as obesity, T2DM, cardiovascular disease, or clinically relevant phenotypic traits.

Fourth, we recruited a larger sample of women with GDM compared to the control group. This limit is because the recruitment was conducted primarily at the Diabetes and Metabolism Unit.

Therefore, future studies are warranted for better understanding the GDM pathogenesis considering gene-gene and gene-environmental interactions.

## Supplementary Material

Supplementary Tables showing genotypes distribution in study subjects and the correlation between SNPs and clinical data (lipid parameters and BMI) of GDM patients.

## Figures and Tables

**Table 1:** Characteristics of cases and controls.

Characteristics	GDM yes (*N* = 102)	GDM no (*N* = 66)	*p* ^*∗*^
Age (years)^a^	34.6 ± 5.4	31.9 ± 5.1	<0.0001
Ethnicity^b^			0.56
Caucasian	99.0	97.0	
Other	1.0	3.0	
School education^b^			0.68
Low school	20.0	14.5	
High school	44.0	49.1	
University degree	36.0	36.4	
Employment^b^			0.11
Employed	56.9	53.6	
Unemployed	42.2	39.3	
Student	1.0	7.1	
Marital status^b^			0.24
Single	17.8	29.5	
Married	81.2	70.5	
Separated/divorced	1.0	0.0	
BMI (Kg/m^2^) prepregnancy^a^	26.0 ± 8.4	21.3 ± 7.4	0.001
Systolic blood pressure (mmHg)^a^	114 ± 14	121 ± 14	0.10
Diastolic blood pressure (mmHg)^a^	72 ± 14	77 ± 15	0.03
Hypertension^b^	7.4	8.3	0.91
Smoking^b^			0.10
No	74.3	72.7	
Yes	2.0	9.1	
Ex	23.7	18.2	
Family history of DM (1st degree)^b^	33.3	17.6	0.09

^*∗*^Mann–Whitney *U* Test or Chi-square test.

^a^Data are presented as means ± SD.

^b^Data are presented as percent (%).

**Table 2:** OGTT values of cases and controls.

OGTT^a^	GDM yes	GDM no
T0	91,89 ± 8,77	79,50 ± 7,26
T60	160,86 ± 32,31	121,70 ± 25,63
T120	146,57 ± 35,42	107,87 ± 19,81

^a^Data are presented as means ± SD.

## Abbreviations

CVD: Cardiovascular diseases
CHD: Coronary heart disease
GDM: Gestational diabetes mellitus
GIP: Glucagon intestinal peptide
GLP: Glucagon-like peptide 1
IADPSG: International association of diabetes and pregnancy study groups
MODY: Maturity onset diabetes of the young
ORs: Odds ratios
OXLDL: Oxidized LDL
SNPs: Single nucleotide polymorphisms
T2DM: Type 2 diabetes mellitus
TG: Triglycerides.
