# Supplementary material for: Molecular Analysis of a Genetic Variants Panel Related to Nutrients and Metabolism: Association with Susceptibility to Gestational Diabetes and Cardiometabolic Risk in Affected Women
Source: J Diabetes Res. 2017 Jan 4;2017:4612623. doi: 10.1155/2017/4612623 (PMC5241477; doi:10.1155/2017/4612623)
Supplement: Supplementary file 1 — Supplementary Tables showing genotypes distribution in study subjects and the correlation between SNPs and clinical data (lipid parameters and BMI) of GDM patients. [file 4612623.f1.pdf]

Supplementary Table 1. Genotypes distribution according to the presence of GDM (data are presented as percent %).

	GDM (N=102)	No GDM (N= 66)	P*
TCF7L2 rs7903146			0.02*
CC	37.3	51.5	
CT	35.3	37.9	
TT	27.4	10.6	
PPARG2 rs1801282			0.79
CC	77.3	75.5	
CG	22.7	24.5	
GG	0.0	0.0	
PPARGC1A rs8192678			0.33
CC	31.4	31.8	
CT	52.9	43.9	
TT	15.7	24.3	
APOA5 rs662799			0.22
TT	55.9	66.7	
CT	38.2	31.8	
CC	5.9	1.5	
MC4R rs17782313			0.23
TT	64.7	63.6	
CT	28.4	34.9	
CC	6.9	1.5	
LDLR rs2228671			0.92
CC	66.7	68.2	
CT	32.4	30.3	
TT	1.0	1.5	
GCKR rs1260326			0.94
CC	22.5	22.7	
CT	56.9	54.5	
TT	20.6	22.7	
FTO rs9939609			0.30
TT	32.4	24.2	
AT	38.2	50.0	
AA	29.4	25.8	
MTHFR rs1801133			0.53
CC	28.5	34.8	
CT	48.0	39.4	
TT	23.5	25.8	
* Chi square test			

Supplementary Table 2. 3rd trimester TC, HDL-C, LDL-C and TG levels of GDM patients according to carrier/no carrier (data are mean±SD).

	TC mg/dl	P*	HDL-C mg/dl	P*	LDL-C mg/dl	P*	TG mg/dl	P*
TCF7L2 rs7903146		0,80		0,39		0,54		0,45
CC	263±54		68±13		133±66		230±82	
CT-TT	263±53		72±17		130±58		237±72	
PPARG2 rs1801282		0,05		0,50		0,02*		0,09
CC	270±54		70±15		137±60		242±76	
CG-GG	244±47		73±20		112±59		208±70	
PPARGC1A rs8192678		0,43		0,28		0,96		0,43
CC	255±52		68±18		133±56		219±54	
CT-TT	268±54		72±15		125±63		239±83	
APOA5 rs662799		0,41		0,02*		0,74		0,10
TT	258±52		74±17		132±55		222±71	
CT-CC	270±55		66±13		130±68		251±80	
MC4R rs17782313		0,41		0,03*		0,92		0,21
TT	259±52		73±16		133±59		226±73	
CT-CC	269±55		65±14		129±65		249±79	
LDLR rs2228671		0,07		0,48		0,01*		0,91
CC	269±51		72±17		146±52		234±79	
CT-TT	250±57		67±13		104±67		235±69	
GCKR rs1260326		0,38		0,12		0,54		0,23
CC	254±56		66±14		141±55		214±54	
CT-TT	266±52		72±16		128±62		241±81	
FTO rs9939609		0,88		0,11		0,02*		0,60
TT	265±61		75±17		108±68		239±72	
AT-AA	262±50		69±15		142±54		232±78	
MTHFR rs1801133		0,51		0,87		0,50		0,57
CC	267±49		69±15		136±70		226±72	
CT-TT	261±55		71±16		130±59		237±77	
* Mann-Whitney U Test								

Supplementary Table 3. 3rd trimester TC, HDL-C, LDL-C and TG levels of GDM patients according to genotypes (data are mean±SD).

	TC mg/dl	P*	HDL-C mg/dl	P*	LDL-C mg/dl	P*	TG mg/dl	P*
TCF7L2 rs7903146		0,68		0,67		0,42		0,69
CC	264±54		68±13		133±66		230±82	
CT	270±59		72±18		139±59		242±80	
TT	253±43		71±17		120±56		230±61	
PPARG2 rs1801282		0,05		0,50		0,02*		0,09
CC	268±54		70±15		137±60		242±76	
CG	244±47		73±20		112±59		209±71	
GG	0.0		0.0		0.00		0.00	
PPARGC1A rs8192678		0,72		0,56		0,37		0,69
CC	255±52		68±18		133±56		219±54	
CT	268±59		72±16		125±69		239±84	
TT	264±40		71±14		149±35		245±82	
APOA5 rs662799		0,317		0,05		0,34		0,12
TT	258±52		74±17		132±55		222±71	
CT	274±56		66±14		135±68		243±75	
CC	243±42		61±8		101±60		302±102	
MC4R rs17782313		0,375		0,02*		0,69		0,35
TT	259±52		73 ±16		133±59		226±73	
CT	266±57		63±13		126±69		256±85	
CC	287±43		76±15		140±76		217±27	
LDLR rs2228671		0,18		0,36		0,02*		0,67
CC	269±51		72±17		146±51		234±80	
CT	251±58		67±13		104±68		237±70	
TT	226±43		85±15		106±76		176±27	
GCKR rs1260326		0,65		0,30		0,61		0,33
CC	254±56		66±14		141±55		214±54	
CT	264±50		73±17		123±67		234±75	
TT	271±58		70±15		142±48		257±92	
FTO rs9939609		0,98		0,18		0,06		0,07
TT	265±61		75±17		108±68		239±72	
AT	264±51		66±13		135±59		253±81	
AA	260±51		17±18		150±48		211±69	
MTHFR rs1801133		0,46		0,81		0,15		0,85
CC	267±49		69±15		136±67		226±72	
CT	266±57		70±16		140±59		237±81	
TT	253±51		73±17		110±66		237±72	
* Kruskall-Wallis one-way ANOVA								

Supplementary Table 4. GDM and controls BMI in according to genotypes (data are mean±SD).

	BMI	P*
TCF7L2 rs7903146		0,12
CC	23,1±9,2	
CT	24,8±7,3	
TT	26,4±6,6	
PPARG2 rs1801282		0,28
CC	24,9±7,1	
CG	22,7±10,8	
GG		
PPARGC1A rs8192678		0,23
CC	25,0±6,7	
CT	23,2±9,2	
TT	26,3±6,7	
APOA5 rs662799		0,93
TT	24,5±8,5	
CT	24,1±7,8	
CC	25,0±6,1	
MC4R rs17782313		0,57
TT	23,9±7,2	
CT	25,0±9,9	
CC	26,9±6,8	
LDLR rs2228671		0,21
CC	24,3±7,9	
CT	25,0±7,9	
TT	11,0±15,5	
GCKR rs1260326		0,95
CC	24,4±9,2	
CT	24,1±8,3	
TT	25,3±6,0	
FTO rs9939609		0,81
TT	23,4±9,0	
AT	24,8±7,1	
AA	24,9±8,3	
MTHFR rs1801133		0,27
CC	22,6±10,2	
CT	25,2±6,6	
TT	25,1±7,3	
* Kruskall-Wallis one-way ANOVA		

Supplementary Table 5. GDM and controls BMI in according to carrier/no carrier (data are mean±SD).

	BMI	P*
TCF7L2 rs7903146		0.05
CC	23,0±9,2	
CT-TT	25,4±7,0	
PPARG2 rs1801282		0,28
CC	24,9±7,1	
CG-GG	22,7±10,8	
PPARGC1A rs8192678		0,46
CC	25,0±6,7	
CT-TT	24,1±8,6	
APOA5 rs662799		0,74
TT	24,5±8,5	
CT-CC	24,2±7,6	
MC4R rs17782313		0,41
TT	23,8±7,1	
CT-CC	25,3±9,5	
LDLR rs2228671		0,49
CC	24,3±7,9	
CT-TT	24,5±8,5	
GCKR rs1260326		0,74
CC	24,4±9,2	
CT-TT	24,4±7,7	
FTO rs9939609		0,86
TT	23,4±9,0	
AT-AA	24,8±7,6	
MTHFR rs1801133		0,11
CC	22,6±10,2	
CT-TT	25,1±6,8	
* Mann-Whitney U Test		
